# Current and Potential Developments of Cortisol Aptasensing towards Point-of-Care Diagnostics (POTC)

**DOI:** 10.3390/s17051180

**Published:** 2017-05-22

**Authors:** Azrul Syafiq Zainol Abidin, Ruslinda A. Rahim, Mohd Khairuddin Md Arshad, Mohd Faudzi Fatin Nabilah, Chun Hong Voon, Thean-Hock Tang, Marimuthu Citartan

**Affiliations:** 1 Institute of Nano Electronic Engineering (INEE), Universiti Malaysia Perlis, Kangar, Perlis 01000, Malaysia; azrulzainol@gmail.com (A.S.Z.A.); mohd.khairuddin@unimap.edu.my (M.K.M.A.); nabilah.faudzi@gmail.com (M.F.F.N.); chvoon@unimap.edu.my (C.H.V.); 2 Advanced Medical & Dental Institute (AMDI), Universiti Sains Malaysia, Kepala Batas, Penang 13200, Malaysia; tangth.amdi@gmail.com (T.-H.T.); citartan@gmail.com (M.C.)

**Keywords:** anxiety, cortisol, aptasensor, aptamer

## Abstract

Anxiety is a psychological problem that often emerges during the normal course of human life. The detection of anxiety often involves a physical exam and a self-reporting questionnaire. However, these approaches have limitations, as the data might lack reliability and consistency upon application to the same population over time. Furthermore, there might be varying understanding and interpretations of the particular question by the participant, which necessitating the approach of using biomarker-based measurement for stress diagnosis. The most prominent biomarker related to stress, hormone cortisol, plays a key role in the fight-or-flight situation, alters the immune response, and suppresses the digestive and the reproductive systems. We have taken the endeavour to review the available aptamer-based biosensor (aptasensor) for cortisol detection. The potential point-of-care diagnostic strategies that could be harnessed for the aptasensing of cortisol were also envisaged.

## 1. Diagnosis of Stress

Stress is defined as the internal process of the body that results in a response to a demand or threat (stressor) that is perceived to exceed the resources available to cope with it [1]. As stress can have many negative consequences such as immune system suppression, elevated risk of heart disease/stroke, aging, and infertility, an accurate diagnosis of stress is warranted [2]. Current strategies to diagnose stress include self-reporting of stress, the measurement of the stress effects, the measurement of stress exposure, and the assessment of biomarkers [3]. Among these strategies, biomarker-based measurement appears to be unequivocal for the effective diagnosis of stress. Efficient interpretation of biomarkers is able to provide excellent diagnosis of stress.

## 2. Cortisol as a Potential Stress Biomarker

The central component of the neuroendocrine system is the hypothalamus-pituitary-adrenal (HPA) axis, which controls the response to stress, digestion, and emotions. In the presence of perceived stress, the hypothalamus, which contains neurons, will secrete vasopressin and corticotrophin-releasing hormone (CRH). This action triggers the production and secretion of adrenocorticotropic hormone (ACTH) from the pituitary gland. Subsequently, the adrenal cortex is stimulated to produce glucocorticoids, primarily cortisol. Cortisol has immunosuppressive and anti-inflammatory effects, increases heart rate and blood pressure, and represses growth, and digestive and reproductive activities. Cortisol is also involved in the maintenance of energetic balance [4]. It also has a function in the mediation of life history trade-offs [5] and allows for individuals to respond to disturbances in their environment [6]. 

The glucocorticoids, including cortisol, repress the production of CRH and ACTH, as a negative feedback response [7]. Since cortisol is the primary end product of the HPA axis, it can be employed as an amenable biomarker for the diagnosis of stress. A significant deviation in the cortisol level from the normal range in sweat, serum, and saliva is found to be associated with stress conditions [8,9,10,11] substantiating the use of cortisol as a suitable biomarker for stress. 

A potential biomarker of chronic disease defined by the recent use of hair cortisol measurements prove that it gives a retrospective index of incorporating cortisol secretion over a period of several months [12,13]. Over the last 20 years, hair sample testing has increased in recognition and attention, especially for forensic purposes and the detection of illegal drugs. This is because hair can keep a relatively long-term record of retrospective levels of various biomarkers in the body [14]. High-performance liquid chromatography (HPLC) ion spray mass spectrometry has been developed for hair cortisol assay [15,16]. Increased hair cortisol in rhesus macaques has been linked with a prolonged stressful experience. The immunoassay for testing hair cortisol has been developed by Davenport et al. [17] during the third trimester of pregnancy. Kirschbaum et al. [18] found that increased cortisol production has been systemically incorporated into growing hair by using the same method. It has been able to provide a retrospective long-term history of cortisol exposure for up to six months. A long-term follow-up study in the same individual can use these findings to indicate the use of hair for sample cortisol levels. It also has a potential for biomarker use in stress applications. Another famous cortisol sample type is by using blood or serum. Even though limited use in the clinical laboratory, serological methods are able to provide high sensitivity and specificity [19].

**Figure 1:**
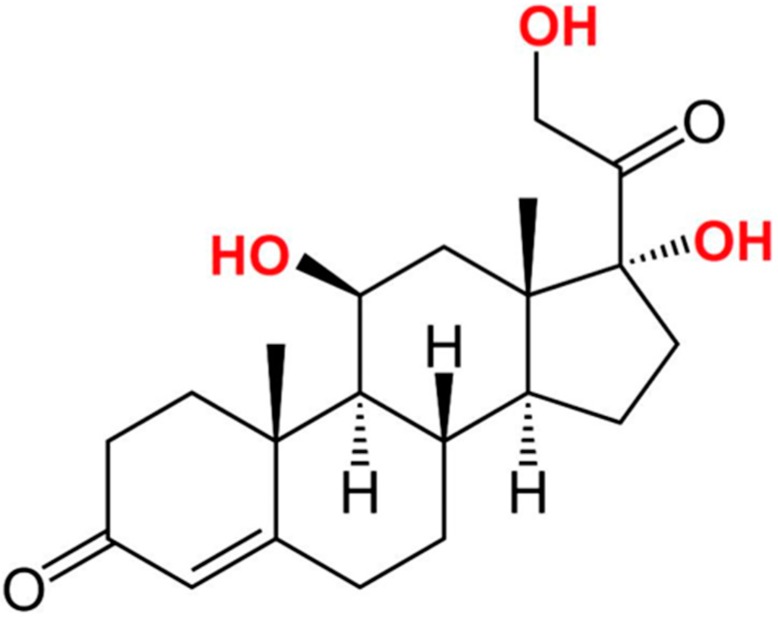
Chemical structure of cortisol. It has the chemical formula C_21_H_30_O_5_ and a molar mass of 362.460 g/mol.

Dispersion to all water spaces of the body, such as saliva, serum, and urine, is the characteristic of cortisol secreted from adrenal glands. Urinary measures have the drawback of the inability to determine rapid changes in the cortisol level. In clinical and research settings, serum measures are often used but the stress from the venipuncture itself elevates the level of cortisol. Moreover, to perform daily serial blood collection is not practical. Contrarily, salivary cortisol samples benefit from easy collection. It is also present in the form of a bioactive fraction, but not in cortisol bound to cortisol-binding globulin (CBG) or other proteins [20]. Using anti-cortisol antibodies, many immune-sensing applications of cortisol have been developed for the diagnostic detection of stress, as summarized in Table 1. The structure of cortisol is illustrated in Figure 1.

## 3. Aptamers as a Potential Diagnostic Element That Can Replace Anti-Cortisol Antibodies

Although anti-cortisol antibodies have been applied in many immune sensing applications of cortisol, antibodies generally have many limitations. Antibodies can be permanently denatured, and the generation of antibodies requires animals for production [28]. Antibodies have batch-to-batch variations [29]. The conjugation of antibodies is difficult and expensive [30]. These shortcomings of antibodies can be addressed by aptamers [31].

Aptamers are single-stranded DNAs or RNAs that have high affinity and specificity against the corresponding target [32]. Aptamers have reversible denaturation properties and are isolated in vitro. Aptamers, unlike antibodies, exhibit no batch-to-batch variation [33]. Owing to the superior properties of aptamers over antibodies, aptamers can be an elegant candidate to replace the counterpart antibodies in diagnostic assays of cortisol.

## 4. In Vitro Isolation of Aptamers Against Cortisol

**Figure 2:**
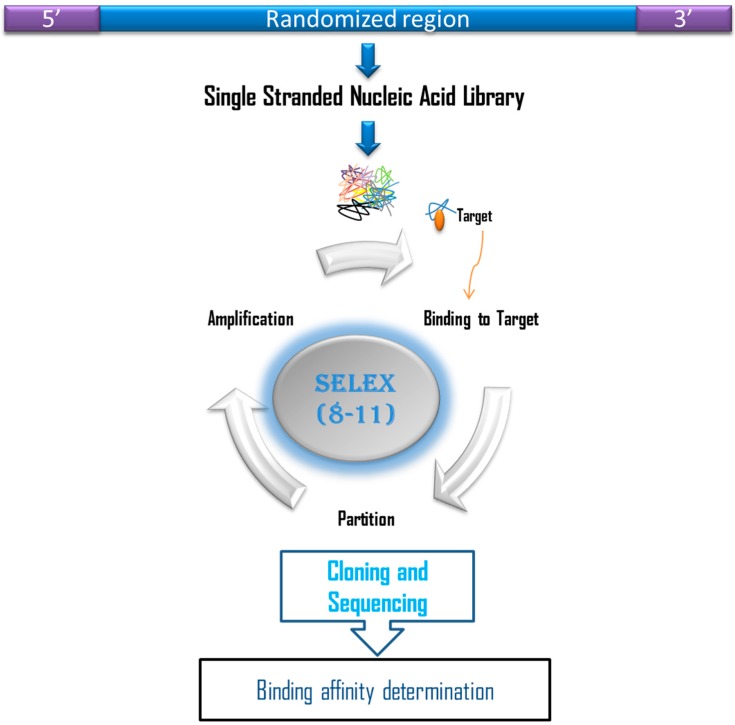
Systematic Evolution of Ligands by EXponential enrichment (SELEX). The binding molecules are selected from a randomized nucleic acid library. Initially, the nucleic acid library is incubated with the target molecule. Then, the unbound nucleic acids are washed away, leaving only the molecules that have bound to the target molecule. The target-bound nucleic acid molecules will be eluted and amplified. Following amplification, the resulting double stranded DNA (dsDNA) will be converted to ssDNA in the case of DNA aptamer generation. For RNA aptamer generation, the dsDNA will be subjected to in vitro *transcription* to generate RNA pool. The resulting ssRNA/DNA pool will be used for the subsequent round of SELEX. Several rounds of SELEX will be carried out till the isolation of nucleic acid molecules that have high affinity and specificity against the target. The binding affinity of the putative aptamers will be estimated.

Cortisol is a very small molecule with a size of 362.46 Daltons. This small size could complicate the conventional process of selecting aptamers by Systematic Evolution of Ligands by EXponential enrichment (SELEX) (Figure 2). Therefore, tweaking of the conventional SELEX is required so that binding sites of the target are available for the interaction with potential aptamers. On this basis, some researchers [34] have designed a tunable stringency magnetic bead selection strategy to generate aptamers against cortisol. This SELEX process was adopted from a previously described method [35] in which aptamers were generated against four standard NTPs. In this method, structure-switching aptamers are directly isolated from the nucleic acid library immobilized on the surface of the magnetic beads, which interacts with the target that is free in the solution. This selection strategy differs significantly from the conventional selection that generates aptamers, which can only interact with the target and most probably are unable to undergo a conformational change that can be transduced into signal production. Martin et al. (2014) immobilized the biotinylated capture probe on the surface of gold nanoparticles. The capture probe was a 7-mer complementary to the 5′-end of the ssDNA library with the sequence GAATGGATCCACATCCATGG-N40-TTCACTGCAGACTTGACGAAGCTTGACGAA. This complementarity allows duplex formation between the capture probe and the ssDNA library. The key to this selection strategy is that, once the target is added, the sequences that have an affinity for the target will be released from the duplex formed initially with the capture probe. These sequences, which are now bound to the target, can be separated from the unbound sequences by magnetic forces. Stringency was gradually increased by increasing the length of the capture probe. This allows only sequences with high affinity to be released from the duplex and bind to the target. Stringency was increased from cycle 1 to cycle 10, but was decreased from cycle 11 to cycle 13 and was increased again to cycle 15. A single sequence of aptamer 15 to 1 was isolated after 15 cycles of SELEX, which demonstrated an equilibrium dissociation constant (K_d_) value of 6.9 ± 2.8 μM, as estimated by equilibrium dialysis, or 16.1 ± 0.6 μM, as estimated by microscale thermophoresis.

The K_d_ value is in accordance with the K_d_ values of aptamers selected against other small molecules such as 17β-estradiol (0.9 µM) [36], kanamycin A (3.9 µM) [37], kanamycin A (2.8 µM) [38], ketamine (0.59 µM) [39], or Pd II (4.60 ± 1.17 μM) [40]. The K_d_ value is in the micromolar range due to the comparatively small size of the target to the aptamer [41]. 

## 5. Cortisol Aptasensor towards Point-of-Care Diagnostics

Point-of-care diagnostic is a type of in vitro diagnosis that enables the analysis of diseases to be carried out anywhere, i.e., from the hospital to the field, for an instant answer to expedite rapid treatment [42]. For a sensor to be used as a point-of-care diagnostic device, it should involve the usage of a small sample volume, an inexpensive disposable platform, microfluidic features to control sample flow, a reasonable reaction time, removal/masking of interfering agents, and an effective measurement strategy [43,44]. To date, there are two applications of cortisol aptasensors. These aptasensing applications potentially fulfill the criteria that qualify them as potential point-of-care devices. Each of these aptasensing applications will be scrutinized to evaluate its capacity as a point-of-care diagnostic device. 

### 5.1. Gold Nanoparticles

**Figure 3:**
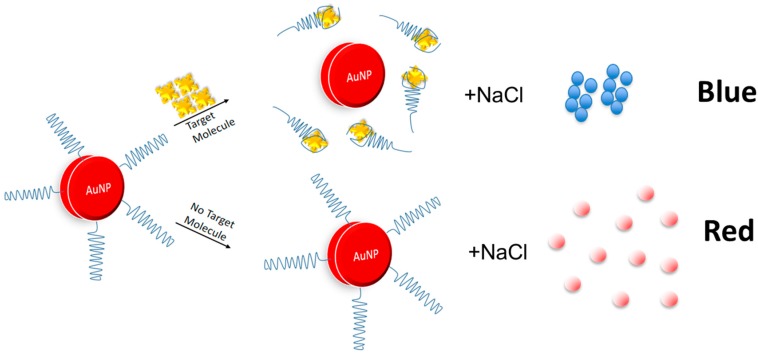
The mechanism of the colorimetric detection of a small target molecule using aptamers. In the presence of the target, aptamers are desorbed from the surface of AuNPs forming aptamer-target complex. As a result, Na^+^ neutralizes the negatively charged citrate ion on the surface of AuNP. This causes aggregation of the AuNPs and results in blue colour formation. In the absence of the target, aptamers on the surface of AuNPs stabilizes these nanoparticles against the NaCl-induced salt aggregation, causes the production of red colour.

One of the ideal assays for any point-of-care diagnostic application is a colorimetric assay that enables detection of the target by the naked eye. One assay that is able to meet this criterion is the gold nanoparticle-based assay. Compared to antibodies, aptamers are more compatible with the gold nanoparticle-based assay due to the differential absorption of the aptamers onto the surface of the gold nanoparticles in the presence and absence of the target, resulting in color changes [45]. Aptamers are absorbed onto the surface of the gold nanoparticles due to the interaction between the nitrogenous bases of the aptamers with gold atoms [46]. In the absence of the target, aptamers are absorbed onto the surface of the gold nanoparticles and stabilize them against the NaCl-induced aggregation. The gold nanoparticles are segregated which causes changes in the surface plasmon of the gold nanoparticles, resulting in red colour production. However, in the presence of the target, aptamers are desorbed from the surface of the gold nanoparticles, thus permitting Na^+^ to neutralize the negatively-charged citrate ions on the surface of the gold nanoparticles. As a consequence, gold nanoparticles are aggregated, which alters the surface plasmon and results in the production of blue color (Figure 3). Based on this principle, aptamer generated against cortisol (Aptamer 15 to 1) was applied in the gold nanoparticle assay, achieving a detection limit of 150–600 nM [34]. The detection limit corresponds to the normal range of free cortisol (30 to 140 ng/mL or 100 to 500 nM) in human serum. The assay developed exhibited remarkable specificity as it was found to be unresponsive to other stress biomarkers, such as epinephrine and norepinephrine, and also non-selective against other structurally-similar molecules of cortisol.

The high sensitivity and selectivity of the aptamer-based gold nanoparticle assay can be attributed to the structure-switching property of the aptamer in the presence or absence of the target [47,48]. Further work was conducted to increase the DNA coverage from 73 DNA molecules per gold nanoparticle (D/NP) to 120 to 200 D/NP. However, the limit of detection diminished as the coverage increased, i.e., 29.5 nM for 73 D/NP, 145.2 nM for 120 D/NP, and 27.3 μM for 200 D/NP [49]. This result was in agreement with studies conducted by Smith et al. (2014), who found that the limit of detection decreased when the cocaine aptamer coverage was increased from 60 aptamers per gold nanoparticle to 300 aptamers per gold nanoparticle [50]. Aptamer-based gold nanoparticles is highly sensitive and specific without the requirement for any expensive instrumentation, suggesting that this method can be a potential point-of-care diagnostic strategy for cortisol detection.

### 5.2. Surface Immobilization-Free Electrochemical Detection of Cortisol

**Figure 4:**
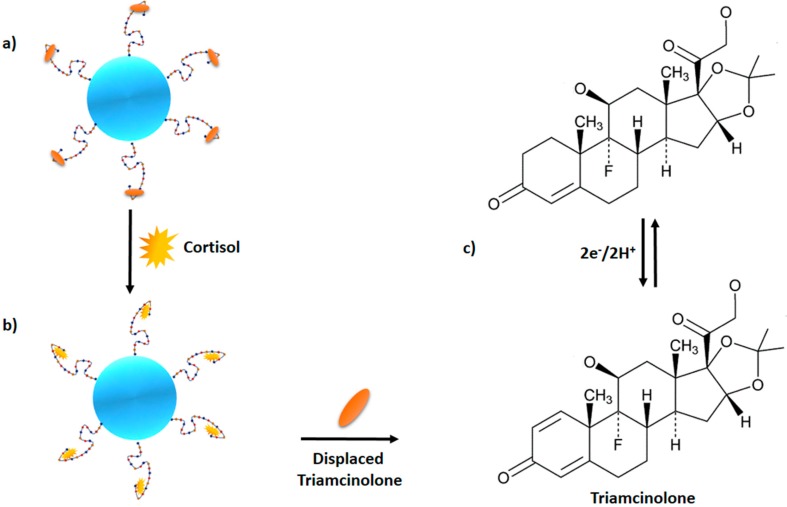
(**a**) Complex of triamcinolone-aptamer-AuNPs, (**b**) cortisol displaces triamcinolone from aptamer-AuNPs, (**c**) triamcinolone are electrochemically reduced on the graphene-modified electrodes producing signal.

An immobilization-free electrochemical sensor can obviate the need for immobilizing the aptamer on the electrode surface, as well as washing to remove the non-specifically-bound target or labeling of the capture probe. In the assay developed by [51], target recognition by the aptamer and signal production are independent and thus, could be optimized separately for better specificity. Figure 4 illustrates the mechanism of surface immobilization-free electrochemical detection of cortisol. The cortisol aptamer conjugated to gold nanoparticles is bound to triamcinolone, which is structurally similar to cortisol. In the presence of cortisol in the sample, cortisol will displace triamcinolone from the aptamer. As such, triamcinolone is electrochemically reduced on graphene-modified electrodes. The graphene-modified glass electrodes were patterned on the coverslip glass by lithography patterning within a nanoslit device. These patterned electrodes serve as the counter, reference and working electrodes. Linearity was achieved within the range of 10 μg/mL to 30 pg/mL without profound interferences from other glucocorticoids such as estradiol, progesterone, and testosterone. The developed assay is similar to ELISA and radiolabeling but involves a shorter assay time (2.5 min) and a smaller sample volume (<1 μL). The small sample volume and faster reaction time are in accordance with the criteria of point-of-care diagnostics, suggesting that surface immobilization-free electrochemical detection of cortisol can be a potential point-of-care diagnostic method for cortisol detection.

## 6. Prophesying Aptamer-Based Lateral Flow Assay (LFA) for Cortisol Detection

**Figure 5:**
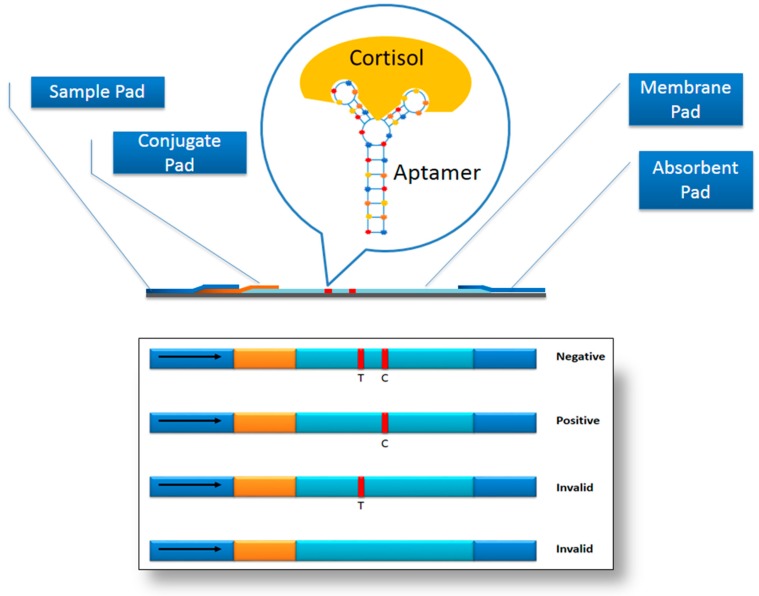
Essential illustration of the LFA integrated with an aptamer.

The lateral flow assay (LFA) is one of the most sought after point-of-care diagnostic devices as it is a mobile system that can provide a quantitative and qualitative measurement of the target. LFA consists of a sample pad, conjugate pad, membrane pad and absorbent pad. The absorbent pad provides the capillary effect for the flow of the sample, while the conjugate pad is usually dry and is dispensed with the labeled molecular recognition elements (MRE). The labeled MRE binds to target in the sample and carries it away. Due to capillary action, the labeled MRE-target complex encounters another MRE immobilized on the surface of the test and control lines on the membrane pad. The formation of the sandwich configuration that consists of the two MREs binding to the target causes the appearance of the coloured visible lines on the test and control zones. The absorbent pad helps to maintain the flow rate of the sample [52]. One format that can be adopted for the design of LFA for the cortisol detection is the competitive format, inspired by the development of LFA for ochratoxin A [53]. The single cortisol aptamer sequence can be extended with homopolymer poly d(A) with thiol at the extremity and immobilized on the surface of AuNPs. In the test zone, a biotinylated probe that is complementary to the aptamer sequence [without the poly d(A)] is immobilized via streptavidin. In the absence of the target, the aptamer-AuNP complex binds the complementary sequence on the test lines, causing the appearance of the visible line. In the presence of the target in the sample, the aptamer-AuNP conjugate will bind to the target and is thus unable to bind to the complementary sequence. As such, there is no visible line on the test zone. To ensure the validity of the test, another oligonucleotide, a poly d(T) oligonucleotide, which is complementary against the poly d(A) is also biotinylated and conjugated via streptavidin onto the surface of the control zone. The intensity of the lines on the test zone is inversely proportional to the amount of the target in the sample. Figure 5 highlights the elements of the LFA.

## 7. Limitation of Aptamer-Based Sensor

As a matter of fact, aptamer degradation (RNA aptamers) by nucleases in blood, and biological media is the main concern that limits their application. Decay of the oligonucleotide takes from several minutes to several tens of minutes depending on the properties of the oligonucleotide [54]. Although aptamers have high specificity, they also bind to the molecules with similar structure. Aptamers isolated against DNA polymerase β can also bind and inhibit DNA polymerase κ, which belongs to another DNA polymerase family. Thus, circumspection should be exercised when adopting aptamers in sensor that might result in false positive signal production. 

## 8. Potential Development of Surface Plasmon Resonance (SPR)-Based Aptasensor

**Figure 6:**
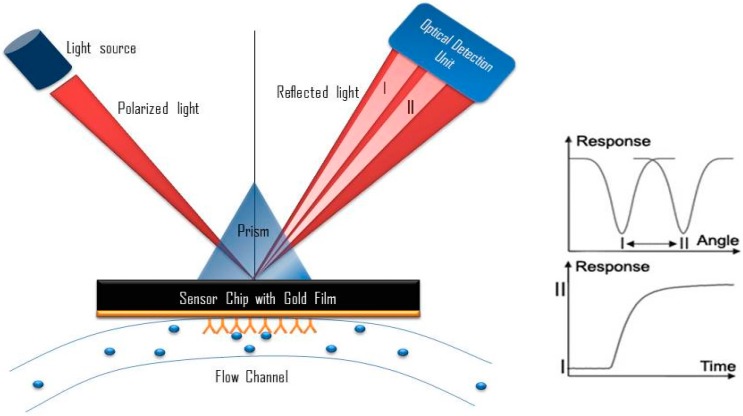
Schematic of the surface plasmon resonance system. The changes in the reflected light I and II influence the response of the system and can be used to determine the specimen. SPR-based aptasensing can be employed for the detection of cortisol. Biotinylated aptamer against cortisol can be immobilized on the surface of the Sensor chip streptavidin (SA). Reflectivity can be used to measure the interaction of the aptamer with the cortisol.

A SPR sensor can be described as a surface-sensitive optical technique to measure the biomolecular interaction based on the changes of the surface plasmon on the metal surface [55,56]. The metal surface of a SPR immunosensor is excited with an incident light at a certain angle of incidence. The immobilization of biomolecules and the subsequent interaction of the biomolecules with the interacting partners cause the change in the reflectivity of the sensing medium. Subsequently, the reflectivity change is measured by the detector, which is exploited to measure the biomolecular interaction. SPR is able to study the biomolecular interaction in real-time in response to the variation of the effective refractive index of the medium [57]. This has been widely used in the field of pharmaceutical development and life sciences [58,59,60]. Moreover, SPR-based affinity measurement could provide rapid, highly selective, safe, and highly sensitive measurements without the need of isotopes or fluorescence labels [61,62]. Figure 6 shows the schematic diagram of the SPR system.

Frasconi et al. have used a SPR-based immunosensor for real-time measurement of cortisone and cortisol levels from samples of urine and saliva. They have used polycarbocylate-hydrogel-based coating to immobilize the antibody. During repeated regeneration and affinity reaction cycles, the sensor surface demonstrates a high level of stability. In addition to showing high specificity for cortisone and cortisol, there is no profound interference from other types of steroids with similar chemical structures. They also investigated the suitability of hydrogen coating, which prevent the nonspecific binding. The proposed method has been correlated with liquid chromatography/tandem mass spectrometry to measure the cortisone and cortisol in urine and saliva samples. The limit of detection is less than 10 μg·L^−1^, which is sensitive enough for applications in clinical and forensic use [30]. Similar application can be adopted to develop SPR-based aptasensing application of cortisol detection. Biotinylated aptamer against cortisol can be immobilized on the surface of the Sensor chip streptavidin (SA). Sample containing cortisol can be injected through the aptamer-immobilized chip and the changes in the reflectivity can be deployed to measure the biomolecular interaction.

## 9. Smartphone-Based Aptasensor

**Figure 7:**
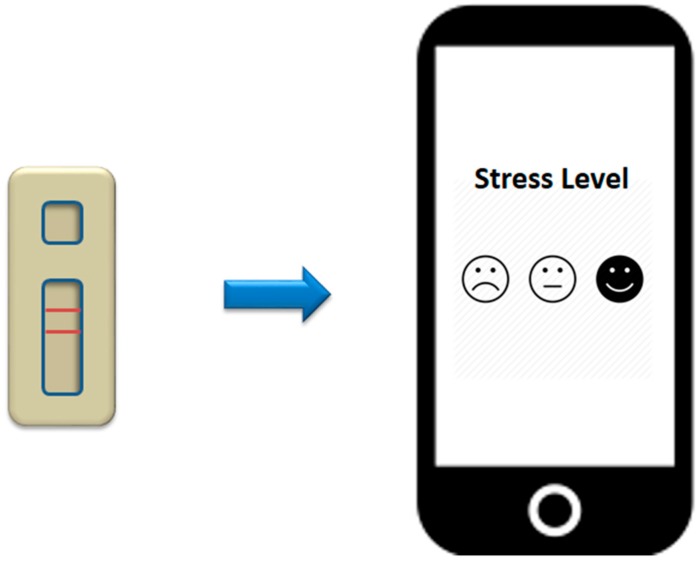
Schematic diagram of the potential smartphone-based cortisol measurement system. Anti-cortisol aptamer can be adopted in the gold nanoparticles-based detection of cortisol and embedded into the smartphone.

A simple, portable, affordable and sensitive sensor is a feasible diagnostic system that is able to meet all the requirements of the ASSURED criteria of a point-of-care diagnostic system. These requirements are inherent in the smartphones, which are portable and simple. Smartphone system can be amalgamated with the label-free way of measuring biomolecular interaction such as gold nanoparticles-based assay. Based on this foundation, the gold nanoparticle-based diagnostic system using cortisol aptamer can be merged with the smartphone system. Inspiration on developing this sensor is acquired from the colorimetric assay-smartphone system developed for the detection of cocaine. The color changes in the presence and absence of the target were precisely measured by using a fully-functional Android-based color analysis application. The color of an unknown sample can be distinguished using this application and compared with positive references to provide a result as to whether the tested substance is positive for cocaine [50]. Another envisaged smartphone-based aptacortisol sensor could be exemplified from the assimilation of the smartphone with the filter-less fluorescent assay using ultraviolet light. The aptamer is conjugated with pyrenes at the two extremities. In the presence of the target, aptamer forms excimer. Due to the biomolecular recognition event, the very large Stokes shift between the emission and the excitation of the excimer is transduced into signal production [63]. Figure 7 highlights the smartphone-based cortisol detection system

## 10. Conclusions

The current development of two cortisol aptasensors has evidenced the potentiality of paving the trajectory towards using anti-cortisol aptamer in the point-of-care diagnostic. Aptamer-based gold nanoparticles assay and surface immobilization-free electrochemical detection of cortisol meet all the requirements of the ASSURED guidelines which are affordable, sensitive, specific, user-friendly, rapid/robust, equipment-free and delivered. POCTs such as LFA, SPR and smartphone system could also lay groundwork towards expending the current repertoire of aptamer-based assay of detecting cortisol. 

## Figures and Tables

**Table 1:** List of antibody-based cortisol sensing mechanisms with the probe, limit of detection (LOD), advantages, and limitations.

Method	Probe	Lowest LOD of Cortisol	Advantage	Limitation	References
*ELISA*	Monoclonal antibody	-	Stable reagents	Uses enzyme labelling	[21]
*Lateral Flow Immunoassays*	Colloidal gold-labelled primary antibody	3.5 μg/L	High sensitivity High selectivity Portable	No washing step Semi-quantitative or Qualitative Uncertain sample volume reduce precision	[22]
*Chemiresistor Immunosensor*	Monoclonal antibody	1 pg/mL	Label-free Rapid detection of cortisol	Non-specific adsorption of mucin from saliva	[23]
*Quartz Crystal Microbalance*	Monoclonal antibody	11 pg/mL	Label free Real time measurement	Measurement noise caused by environment	[24]
*Surface Plasmon Resonance (SPR)*	Monoclonal antibody	10 μg/L	Real time measurement Rapid Safe High-selectivity High-sensitivity	Pre-treatment procedure to get better result	[25]
*Interdigitated µ-Electrode*	Monoclonal antibody	1 pM	Ultrasensitive label-free Impedimetric biosensor	Potential of superimposed effects of multi-electrode measurement	[26]
*Polyaniline protected gold nanoparticles (PPAuNPs)*	Monoclonal antibody	1 pM	Improvement in redox stability and electro-activity	pH might affect the outcome	[27]

## References

[1] Kaplan S. (1995). The restorative benefits of nature: Toward an integrative framework. J. Environ. Psychol..

[2] Cacioppo J.T., Tassinary L.G., Berntson G. (2007). The Handbook of Psychophysiology.

[3] Holleman M., Vreeburg S.A., Dekker J.J.M., Penninx B.W.J.H. (2012). The relationships of working conditions, recent stressors and childhood trauma with salivary cortisol levels. Psychoneuroendocrinology.

[4] Landys M.M., Ramenofsky M., Wingfield J.C. (2006). Actions of glucocorticoids at a seasonal baseline as compared to stress-related levels in the regulation of periodic life processes. Gen. Comp. Endocrinol..

[5] Crespi E.J., Williams T.D., Jessop T.S., Delehanty B. (2013). Life history and the ecology of stress: How do glucocorticoid hormones in fl uence life-history variation in animals?. Funct. Ecol..

[6] Wingfield J.C. (2013). Ecological processes and the ecology of stress: The impacts of abiotic environmental factors. Funct. Ecol..

[7] Varghese P., Brown S. (2001). The Hypothalamic-Pituitary Adrenal Axis in Major Depressive Disorder: A Brief Primer for Primary Care Physicians Femina. Prim. Care Companion J. Clin. Psychiatry.

[8] Delahanty D.L., Raimonde A.J., Spoonster E. (2000). Initial posttraumatic urinary cortisol levels predict subsequent PTSD symptoms in motor vehicle accident victims. Biol. Psychiatry.

[9] McEwen B.S. (2002). Editorial: Cortisol, Cushing’s syndrome, and a shrinking brain—New evidence for reversibility. J. Clin. Endocrinol. Metab..

[10] Morgan C.A., Wang S., Rasmusson A., Hazlett G., Anderson G., Charney D.S. (2001). Relationship among plasma cortisol, catecholamines, neuropeptide Y, and human performance during exposure to uncontrollable stress. Psychosom. Med..

[11] Yehuda R., Halligan S.L., Bierer L.M. (2001). Relationship of parental trauma exposure and PTSD to PTSD, depressive and anxiety disorders in offspring. J. Psychiatr. Res..

[12] Stalder T., Steudte S., Alexander N., Miller R., Gao W., Dettenborn L., Kirschbaum C. (2012). Cortisol in hair, body mass index and stress-related measures. Biol. Psychol..

[13] Russell E., Koren G., Rieder M., Van Uum S. (2012). Hair cortisol as a biological marker of chronic stress: Current status, future directions and unanswered questions. Psychoneuroendocrinology.

[14] Nakahara Y. (1999). Hair analysis for abused and therapeutic drugs. J. Chromatogr. B Biomed. Sci. Appl..

[15] Cirimele V., Kintz P., Dumestre V., Goulle J.P., Ludes B. (2000). Identification of ten corticosteroids in human hair by liquid chromatography-ion spray mass spectrometry. Forensic Sci. Int..

[16] Raul J.S., Cirimele V., Ludes B., Kintz P. (2004). Detection of physiological concentrations of cortisol and cortisone in human hair. Clin. Biochem..

[17] Davenport M.D., Tiefenbacher S., Lutz C.K., Novak M.A., Meyer J.S. (2006). Analysis of endogenous cortisol concentrations in the hair of rhesus macaques. Gen. Comp. Endocrinol..

[18] Kirschbaum C., Tietze A., Skoluda N., Dettenborn L. (2009). Hair as a retrospective calendar of cortisol production-Increased cortisol incorporation into hair in the third trimester of pregnancy. Psychoneuroendocrinology..

[19] Van Doorn H.R., Koelewijn R., Hofwegen H., Gilis H., Wetsteyn J.C.F.M., Wismans P.J., Sarfati C., Vervoort T., Van Gool T. (2007). Use of enzyme-linked immunosorbent assay and dipstick assay for detection of Strongyloides stercoralis infection in humans. J. Clin. Microbiol..

[20] Gozansky W., Lynn J., Laudenslager M., Kohrt W. (2005). Salivary cortisol determined by enzyme immunoassay is preferable to serum total cortisol for assessment of dynamic hypothalamic-pituitary-adrenal axis activity. Clin. Endocrinol..

[21] Sink T.D., Lochmann R.T., Fecteau K.A. (2008). Validation, use, and disadvantages of enzyme-linked immunosorbent assay kits for detection of cortisol in channel catfish, largemouth bass, red pacu, and golden shiners. Fish Physiol. Biochem..

[22] Posthuma-Trumpie G.A., Korf J., van Amerongen A. (2009). Lateral flow (immuno)assay: Its strengths, weaknesses, opportunities and threats. A literature survey. Anal. Bioanal. Chem..

[23] Tlili C., Myung N.V., Shetty V., Mulchandani A. (2011). Label-free, chemiresistor immunosensor for stress biomarker cortisol in saliva. Biosens. Bioelectron..

[24] Ito T., Aoki N., Kaneko S., Suzuki K. (2014). Highly sensitive and rapid sequential cortisol detection using twin sensor QCM. Anal. Methods.

[25] Frasconi M., Mazzarino M., Botrè F., Mazzei F. (2009). Surface plasmon resonance immunosensor for cortisol and cortisone determination. Anal. Bioanal. Chem..

[26] Arya S.K., Chornokur G., Venugopal M., Bhansali S. (2010). Antibody functionalized interdigitated μ-electrode (IDμE) based impedimetric cortisol biosensor. Analyst.

[27] Arya S.K., Dey A., Bhansali S. (2011). Polyaniline protected gold nanoparticles based mediator and label free electrochemical cortisol biosensor. Biosens. Bioelectron..

[28] Sheriff M.J., Dantzer B., Delehanty B., Palme R., Boonstra R. (2011). Measuring stress in wildlife: Techniques for quantifying glucocorticoids. Oecologia.

[29] O’Sullivan C.K. (2002). Aptasensors—The future of biosensing?. Anal. Bioanal. Chem..

[30] Eldin P., Pauza M.E., Hieda Y., Lin G., Murtaugh M.P., Pentel P.R., Pennell C.A. (1997). High-level secretion of two antibody single chain Fv fragments by Pichia pastoris. J. Immunol. Methods.

[31] Mairal T., Ozalp V.C., Lozano Sánchez P., Mir M., Katakis I., O′Sullivan C.K. (2008). Aptamers: Molecular tools for analytical applications. Anal. Bioanal. Chem..

[32] Ellington A.D., Szostak J.W. (1990). In vitro selection of RNA molecules that bind specific ligands. Nature.

[33] Smith J.E., Medley C.D., Tang Z., Shangguan D., Lofton C., Tan W. (2007). Aptamer-conjugated nanoparticles for the collection and detection of multiple cancer cells. Anal. Chem..

[34] Martin J.A., Chávez J.L., Chushak Y., Chapleau R.R., Hagen J., Kelley-Loughnane N. (2014). Tunable stringency aptamer selection and gold nanoparticle assay for detection of cortisol. Anal. Bioanal. Chem..

[35] Nutiu R., Li Y. (2003). Structure-switching signaling aptamers. J. Am. Chem. Soc..

[36] Vanschoenbeek K., Vanbrabant J., Hosseinkhani B., Vermeeren V., Michiels L. (2015). Aptamers targeting different functional groups of 17β-estradiol. J. Steroid Biochem. Mol. Biol..

[37] Stoltenburg R., Nikolaus N., Strehlitz B. (2012). Capture-SELEX: Selection of DNA aptamers for aminoglycoside antibiotics. J. Anal. Methods Chem..

[38] Nikolaus N., Strehlitz B. (2014). DNA-aptamers binding aminoglycoside antibiotics. Sensors.

[39] Sun G.B., Sun H., Meng X.B., Hu J., Zhang Q., Liu B., Wang M., Xu H.B., Sun X.B. (2014). Aconitine-induced Ca^2+^ overload causes arrhythmia and triggers apoptosis through p38 MAPK signaling pathway in rats. Toxicol. Appl. Pharmacol..

[40] Cho Y.S., Lee E.J., Lee G.H., Hah S.S. (2015). Aptamer selection for fishing of palladium ion using graphene oxide-adsorbed nanoparticles. Bioorg. Med. Chem. Lett..

[41] McKeague M., Derosa M.C. (2012). Challenges and opportunities for small molecule aptamer development. J. Nucleic Acids.

[42] Jung W., Han J., Choi J.W., Ahn C.H. (2014). Point-of-care testing (POCT) diagnostic systems using microfluidic lab-on-a-chip technologies. Microelectron. Eng..

[43] Jönsson C., Aronsson M., Rundström G., Pettersson C., Mendel-Hartvig I., Bakker J., Martinsson E., Liedberg B., MacCraith B., Ohman O. (2008). Silane-dextran chemistry on lateral flow polymer chips for immunoassays. Lab Chip.

[44] Wang J., Ahmad H., Ma C., Shi Q., Vermesh O., Vermesh U., Heath J. (2010). A self-powered, one-step chip for rapid, quantitative and multiplexed detection of proteins from pinpricks of whole blood. Lab Chip.

[45] Soh J.H., Lin Y., Rana S., Ying J.Y., Stevens M.M. (2015). Colorimetric Detection of Small Molecules in Complex Matrixes via Target-Mediated Growth of Aptamer-Functionalized Gold Nanoparticles. Anal. Chem..

[46] Wang J., Wang L., Liu X., Liang Z., Song S., Li W., Li G., Fan C. (2007). A gold nanoparticle-based aptamer target binding readout for ATP assay. Adv. Mater..

[47] Slavkovic S., Altunisik M., Reinstein O., Johnson P.E. (2015). Structure-affinity relationship of the cocaine-binding aptamer with quinine derivatives. Bioorg. Med. Chem..

[48] Xie S., Walton S.P. (2009). Application and analysis of structure-switching aptamers for small molecule quantification. Anal. Chim. Acta.

[49] Martin J.A., Smith J.E., Warren M., Chávez J.L., Hagen J.A., Kelley-Loughnane N. (2015). A method for selecting structure-switching aptamers applied to a colorimetric gold nanoparticle assay. J. Vis. Exp..

[50] Smith J.E., Griffin D.K., Leny J.K., Hagen J.A., Chávez J.L., Kelley-Loughnane N. (2014). Colorimetric detection with aptamer-gold nanoparticle conjugates coupled to an android-based color analysis application for use in the field. Talanta.

[51] Sanghavi B.J., Moore J.A., Chávez J.L., Hagen J.A., Kelley-Loughnane N., Chou C.F., Swami N.S. (2016). Aptamer-functionalized nanoparticles for surface immobilization-free electrochemical detection of cortisol in a microfluidic device. Biosens. Bioelectron..

[52] Chen A., Yang S. (2015). Replacing antibodies with aptamers in lateral flow immunoassay. Biosens. Bioelectron..

[53] Zhou W., Kong W., Dou X., Zhao M., Ouyang Z., Yang M. (2016). An aptamer based lateral flow strip for on-site rapid detection of ochratoxin A in Astragalus membranaceus. J. Chromatogr. B Anal. Technol. Biomed. Life Sci..

[54] Lakhin A.V., Tarantul V.Z., Gening L.V. (2013). Aptamers: Problems, solutions and prospects. Acta Nat..

[55] Lioubashevski O., Chegel V.I., Patolsky F., Katz E., Willner I. (2004). Enzyme-catalyzed bio-pumping of electrons into Au-nanoparticles: A surface plasmon resonance and electrochemical study. J. Am. Chem. Soc..

[56] Tokareva I., Minko S., Fendler J., Hutter E. (2004). Polymer Brushes and Gold Nanoparticle Enhanced Transmission Surface Plasmon. J. Am. Chem. Soc..

[57] Homola J. (2006). Electromagnetic Theory of Surface Plasmons. Surface Plasmon Resonnance Based Sensors.

[58] Cooper M.A. (2002). Optical biosensors in drug discovery. Nat. Rev. Drug Discov..

[59] Giannetti A.M., Koch B.D., Browner M.F. (2008). Surface plasmon resonance based assay for the detection and characterization of promiscuous inhibitors. J. Med. Chem..

[60] Lokate A.M.C., Beusink J.B., Besselink G.A.J., Pruijn G.J.M., Schasfoort R.B.M. (2007). Biomolecular interaction monitoring of autoantibodies by scanning surface plasmon resonance microarray imaging. J. Am. Chem. Soc..

[61] Hoa X.D., Kirk A.G., Tabrizian M. (2007). Towards integrated and sensitive surface plasmon resonance biosensors: A review of recent progress. Biosens. Bioelectron..

[62] Homola J. (2008). Surface plasmon resonance sensors for detection of chemical and biological species. Chem. Rev..

[63] Goertz J.P., White I.M. Smartphone-enabled filterless fluorescence assay utilizing the pyrene excimer. Proceedings of the Optics and Biophotonics in Low-Resource Settings.

